# Large-bodied gastric spirurids (Nematoda, Spirurida) predict structure in the downstream gastrointestinal helminth community of wild spiny mice (*Acomys dimidiatus*)

**DOI:** 10.1017/S0031182024000891

**Published:** 2024-07

**Authors:** Jerzy M. Behnke, Joseph A. Jackson, Francis Gilbert, Eman M. E. Mohallal, Anna Bajer

**Affiliations:** 1 School of Life Sciences, University of Nottingham, University Park, Nottingham NG7 2RD, UK; 2 School of Science, Engineering and Environment, University of Salford, Manchester M5 4WT, UK; 3 The Ecology Unit of Desert Animals, Desert Research Centre, 1 Mataf El Matareya St, El Matareya, Cairo, Egypt; 4 Department of Eco-Epidemiology of Parasitic Diseases, Faculty of Biology, Institute of Developmental Biology and Biomedical Sciences, University of Warsaw, Miecznikowa 1, 02-096 Warsaw, Poland

**Keywords:** Acanthocephala, *Acomys dimidiatus*, arthropod-mediated transmission, associations and interactions between helminth species, Cestoda, Nematoda, Oxyuroidea, spiny mice, Spirurida

## Abstract

The dominant helminths infecting spiny mice (*Acomys dimidiatus*) in the montane wadis of the Sinai Peninsula of Egypt are spirurid nematodes, notably *Protospirura muricola* and *Mastophorus muris*. Both are relatively large robust stomach worms that accumulate in hosts resulting in high worm burdens. To ascertain whether the presence of spirurid worms or their burdens alters the host's likelihood of infection with other helminth species, we analysed a database containing quantitative data on helminth parasites of these mice (*n* = 431). This comprised of worm burdens recorded during 4 surveys, conducted at 4-year intervals, in 4 wadis, during late summer of each year. The presence of spirurid worms did not significantly alter species richness with other helminth species nor the likelihood of mice carrying other nematode species. However, there was a significant association, particularly of *P. muricola*, with the presence of intestinal stages of cestodes, and with the acanthocephalan *Moniliformis acomysi*. After controlling for intrinsic and extrinsic factors, mice harbouring spirurid worms had greater worm burdens of other helminths compared with mice without spirurids. Moreover, spirurid worm burdens showed a significant positive covariation with similarly adjusted species richness of other helminths, non-spirurid helminths, non-spirurid nematodes, oxyuroid nematodes and intestinal stage cestode worm burdens. We interpret these results as an indication that the key driver for co-occurrence of spirurids with other helminths is likely to be transmission *via* common arthropod hosts (for cestodes and acanthocephalans), but also that mice carrying the heavier spirurid worm burdens become more susceptible to directly transmitted nematodes such as the Oxyuroidea.

## Graphical abstract

**Figure:**
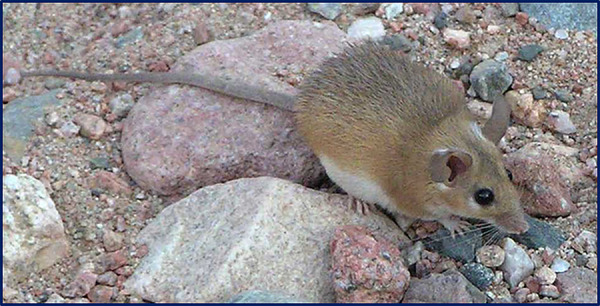

## Introduction

In terms of their prevalence and abundance, spirurid nematodes (Nematoda: Spirurida) are the dominant helminths infecting spiny mice (*Acomys dimidiatus*) inhabiting the dry montane wadis of the Egyptian Sinai Peninsula (Behnke *et al*., 2019). Five species have been identified, all stomach worms, but 2 of the most frequently encountered are *Protospirura muricola* Gedoelst, 1916 and *Mastophorus muris* (Gmelin, 1790) (Table 1). Both are robust, large worms, with individual females measuring up to 5 cm in length and weighing up to 50 mg wet weight (Behnke *et al*., 2000; Smales *et al*., 2009). In their definitive hosts, these species live in the lumen of the stomach, probably feed on stomach contents and both are long-lived (Lowrie *et al*., 2004), accumulating in their rodent hosts with increasing host age (Behnke *et al*., 2004, 2019). Heavy worm burdens, often exceeding 20 mature worms, cause distension of the stomach walls, and in experimental studies in laboratory mice have been found to cause loss of both host and stomach weight (Lowrie *et al*., 2004). In 1 naturally infected spiny mouse, the total biomass (17 worms weighing collectively 392.2 mg) was recorded as equating to 0.87% of the body weight of its host (Behnke *et al*., 2000).

**Table 1.** Overall prevalence and abundance of helminths recovered from *Acomys dimidiatus* in 4 study sites in the Sinai Peninsula during 4 surveys at 4-year intervals

	Prevalence		Abundance	
Taxon/species	% infected	CL_95_	Mean ±	s.e.m.
Helminths (all species)	91.2	86.77–94.21	37.5	7.17
Nematoda (all species)	89.3	84.70–92.79	34.9	7.12
Spirurida (all species)	69.6	63.46–75.16	11.8	1.15
*Protospirura muricola*	52.9	46.50–59.31	10.5	1.14
*Mastophorus muris*	17.4	12.97–22.82	0.68	0.104
*P. muricola* + *M. muris*	64.7	58.37–70.66	11.2	1.14
*Streptopharagus* spp.	16.9	12.52–22.33	0.52	0.105
*Gongylonema aegypti*	4.2	2.18–7.53	0.07	0.021
*Pterygodermatites witenbergi*	0.2^a^	0.05–2.01	<0.01	
Combined NSpir helminths	74.0	68.09–79.33	25.7	7.01
Combined NSpir nematodes	64.0	57.67–70.01	23.1	6.99
Oxyuroidea (all species)	61.0	54.63–67.01	22.1	6.98
*Dentostomella kunzi*	40.1	33.94–46.53	1.8	0.18
*Aspiculuris africana*	21.6	16.70–27.24	0.8	0.12
*Syphacia minuta*	27.1	21.81–33.26	19.5	6.97
Cestoda (all species)	16.5	12.16–21.76	1.7	0.52
Cestoda, intestinal stages	13.9	9.96–18.89	0.41	0.080
*Rodentolepis negevi*	10.2	6.85–14.74	0.31	0.073
*Rodentolepis fraterna*	1.2	0.32–3.58	0.01	0.007
*Mathevotaenia rodenticum*	1.6	0.57–4.25	0.07	0.031
*Witenbergitaenia sinaica*	0.2	0.05–2.01	0.01	0.007
Cestoda, larval stages	2.8	1.28–5.78	1.3	0.52
Acanthocephala				
*Moniliformis acomysi*	5.6	3.26–9.25	0.90	0.408

Most of the data in this table were included in the text in Behnke *et al*. (2019).

a The single worm recovery of *P. witenbergi* has been included in the spirurid nematodes. For explanation, see text.

The other taxon of nematodes showing high prevalence in spiny mice was the Oxyuroidea, comprising 3 species: the oxyurid, *Syphacia minuta* Greenberg, 1969 and the heteroxynematids *Aspiculuris africana* Quentin, 1966 and *Dentostomella kunzi* Myers, 1961 (Table 1). These live in specific, mostly non-overlapping, regions of the intestinal tract, i.e. caecum, large intestine and small intestine, respectively. Females of the largest of these worms, *D. kunzi*, seldom exceed 3 mg wet weight and measure up to 28 mm in length (Behnke *et al*., 2000; but see Myers, 1961). Although worm burdens with *S. minuta* can be very high (>70 worms), maximum burdens with *D. kunzi* and *A. africana* are generally lower (<25 worms), and both species are small by comparison with *P. muricola* and *M. muris* (Behnke *et al*., 2000, 2019).

There has been considerable recent interest in whether different species of parasites interact positively or negatively in their hosts, and hence whether they represent interactive communities with elements of structure arising from between-species interactions (Behnke *et al*., 2001, 2005, 2009*b*; Cattadori *et al*., 2008; Fenton *et al*., 2014; Clerc *et al*., 2019; Dallas *et al*., 2019; Lewis *et al*., 2023). Given the robust, large size of the 2 dominant spirurids, the heavy worm burdens carried by a proportion of naturally infected spiny mice and evidence from experiments in laboratory mice that high worm burdens cause loss of stomach and host weight, we might expect consequences for other concurrently resident species. Therefore, we hypothesized that a possible consequence of carrying spirurid worm burdens, and the resulting stress on host physiology, may be loss of resistance to other helminths. In this paper, we explore associations of spirurid nematodes with other helminth species based on presence/absence (P/A) data, as well as quantitative relationships based on covariance of spirurid worm burdens, and spirurid biomass, with the worm burdens of other helminth species.

## Materials and methods

### Study sites and source of data

The analysis that follows utilized the database of Behnke *et al*. (2019). These authors reported on the long-term spatiotemporal stability and changes in the helminth communities of spiny mice from 4 study sites (Wadis El Arbaein, Gebal, Gharaba and Tlah) in the montane region of the Sinai Peninsula of Egypt. The animals were trapped during 4 surveys conducted at 4-year intervals (2000, 2004, 2008 and 2012) in late summer (August–September) in each of these years. Descriptions of the sites, the methodology used in fieldwork and the subsequent laboratory analysis of worm burdens have all been described comprehensively in Behnke *et al*. (2000, 2004, 2019). Based on morphometric data collected at autopsy and dried eye lens weight, the animals were allocated to 2 age classes (age classes 1 and 2, corresponding to immature and mature mice, respectively; Behnke *et al*., 2004) and were sexed (male or female).

### Definition of terms employed

This paper focuses on the effect of combined spirurids (all 5 species, plus the single isolate of *Pterygodermatites witenbergi* [Rictularioidea] which was also recovered from the stomach), and of the most prevalent of the Spirurida, *P. muricola*, on other helminth taxa. We refer to all other helminth species, except for the Spirurida, as NSpir helminths (all non-spirurid worms), NSpir helminth species richness (all non-spirurid species) and NSpir nematodes (all nematodes other than the Spirurida). For the cestodes we refer to all cestodes (all cestode species and individual worms, both immature and mature intestinal strobilate stages and body cavity or tissue dwelling larvae), intestinal cestodes (immature and mature intestinal stages with proglottids) and larval cestodes (immature stages found in the tissues or body cavities). For further details of all the species involved, see Table 1, and for information on variation of species richness, prevalence and abundance over time, by site and between the sexes and age classes, see Behnke *et al*. (2019).

### Statistical analysis

For data subsets, prevalence values (% of mice infected with the helminth species referred to) are given with 95% confidence limits (CL_95_) in the tables and text and were calculated using bespoke software based on the tables of Rohlf and Sokal (1995). For quantitative data (i.e. abundance as reflected in worm burdens), we provide mean values ± standard error of the mean (s.e.m.) and refer to abundance as defined by Bush *et al*. (1997) and Margolis *et al*. (1982), and derived for all mice in a data subset, including those uninfected by the parasite in question.

Prevalences were analysed using maximum-likelihood techniques based on log-linear analysis of contingency tables in the software package IBM SPSS, version 28 (Armonk, NY, USA). Full factorial models included sex (2 levels, male and female mice), age (2 levels, juvenile and mature mice), geographical locality (4 levels corresponding to the 4 wadis as specified above) and year of survey (2000, 2004, 2008 and 2012), and these are referred to as the 4 factors. Models also included the P/A of infection with a specified parasite taxon. This approach is based on categorical values of factors of interest, which are used not only to fit hierarchical log-linear models to multidimensional cross-tabulations using an iterative proportional-fitting algorithm but also to detect associations between the fitted factors. Multifactorial models were fitted as described previously (Behnke *et al*., 2019), beginning with the full factorial model, followed by simplification of the model until only significant terms remained. Thus, for each level of analysis in turn, beginning with the most complex model, involving all possible main effects and interactions, those combinations that did not contribute significantly to explaining variation in the data were eliminated in a stepwise fashion beginning with the highest-level interaction (backward selection procedure). A minimum sufficient model (MSM) was then obtained, for which the likelihood ratio (LR) of chi-square was not significant, indicating that the model was sufficient in explaining the data. The importance of each term in interactions involving the P/A of a specified taxon in the final model was assessed by the probability that its exclusion would alter the model significantly and these values are given in the text, assessed by an LR test between models with and without each term of interest.

For analysis of quantitative data, we first fitted full factorial generalized linear (GLM) statistical models in R (version 4.3.2, R Core Development Team) with both extrinsic (year and site) and intrinsic (sex and age) factors for all dependent variables, testing models that were based on negative binomial or Poisson error structures, as relevant. The dependent variables were worm burdens of higher taxa (e.g. NSpir helminths, NSpir nematodes, all cestodes, etc. and helminth species richness of all taxa other than the spirurids) as specified in the Results section. We used the ‘step’ command in R to refine these to MSMs. Next, we combined the MSMs with the addition of P/A of spirurid worms or P/A *P. muricola* as factors at 2 levels (present or absent). To evaluate whether worm burdens of other taxa differed significantly between mice with/without spirurids or with/without *P. muricola* we compared MSMs with and without (P/A) spirurids, or with/without *P. muricola*. To test whether increasing worm burdens of specified taxa were associated with increasing worm burdens of spirurids or *P. muricola* (i.e. whether there is quantitative covariance between the worm burdens of these taxa), we combined MSMs of those taxa with either MSMs of spirurid or *P. muricola* worm burdens, the latter fitted as covariates. In R, we also examined linear covariance of the residuals of MSMs of relevant taxa and tested whether slopes (*β* ± s.e.m.) were positive and whether they differed significantly from zero. Figures of the resulting relationships were constructed in the *ggplot2* and *sjPlot* packages of R. Values of LRs for models based on negative binomial errors and deviances (Dev) for models with Poisson errors are provided, and for models with covariates we also provide gradients (*β* + s.e.m), *z* values and then probabilities (*P*) that the slopes differ from zero.

We applied the covariance distribution test of Haukisalmi and Henttonen (1998) which focuses on the frequency distribution of positive and negative covariances (cov) between all pairs of species in a community. We first log_10_ + 1 transformed worm counts, and then obtained significance values from assessment of the number of times the observed covariance for each pair of species was less than or greater than that derived from each of 5000 cycles of randomization of the data. The test was then repeated, but this time with the factor groups (in this case: year, site and host age and sex) taken into consideration (i.e. subsample constraints).

## Results

### Overall prevalence and abundance of helminths in spiny mice

Table 1 provides the overall prevalence and mean worm burdens of the helminths recovered from *A. dimidiatus*, with data combined across the 4 study sites, 4 surveys, both sexes and both age classes. As reported in Behnke *et al*. (2019), collectively nematodes accounted for most of the helminths recovered (93.1% of all helminths were nematodes). For a breakdown of these data by the 4 factors and relevant analyses, see Behnke *et al*. (2019).

**Figure 1.**
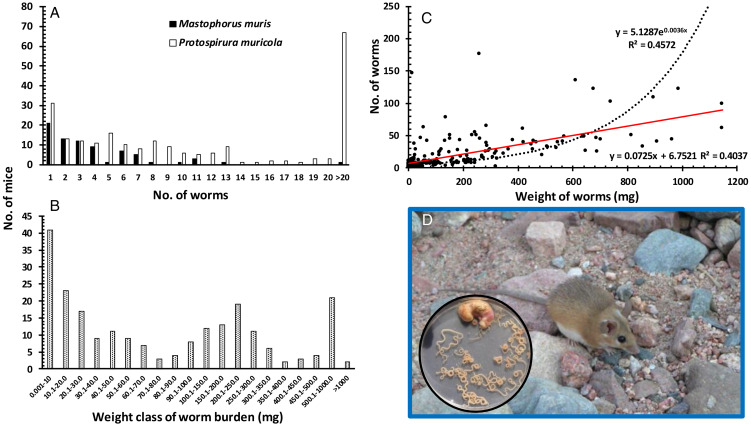
Frequency distribution of *Protospirura muricola* and *Mastophorus muris* worm burdens (A) and their combined biomass (B), the correlation between combined worm burdens and biomass (C) and an adult eastern (Egyptian) spiny mouse (*Acomys dimidiatus*) with inset a spiny mouse stomach containing over 30, mostly mature, *P. muricola* (D). Note that in (B), the abscissa is discontinuous, showing intervals of 10 mg in the mass classes nearest to the ordinate, then intervals of 50 mg and 500 mg, and in (D), the mouse and stomach are approximately of the same scale, the diameter of the standard size Petri dish containing the worms is 9.0 cm. The average body length (nose to anus) of adult mice was 111.7 ± 0.40 mm (*n* = 273), tail length 109.5 ± 0.93 (*n* = 206) and weight 39.7 ± 0.42 g (*n* = 272).

The most prevalent species was *P. muricola*, and this species, with *M. muris*, were the bulkiest and heaviest of the stomach worms. *Mastophorus muris* worm burdens were mostly fewer than 14 worms/mouse, although 1 mouse harboured 27 worms (Fig. 1A). In contrast, many of the mice harboured heavy worm burdens with *P. muricola*; 67 had more than 20 worms, 8 of which had more than 100 worms and the most heavily infected mouse harboured 227 worms of this species. In terms of biomass, 21 mice carried between 500 and 1000 mg of spirurid biomass (Fig. 1B; *P. muricola* and *M. muris* combined), and from 2 mice we recovered a spirurid biomass exceeding 1 g (1.146 and 1.144 g accounting for 3.0 and 2.9% of total body weight of these animals, which carried 99 and 62 *P. muricola*, respectively). There was a positive relationship between the total spirurid biomass carried by mice and the number of spirurid worms recovered, best accounted for by an exponential curve (Fig. 1C; *R*^2^ = 0.457).

### Does the presence of spirurids affect prevalence of other helminths?

The prevalence of NSpir helminths was very similar in mice with [75.3% (70.52–79.59%)] and without spirurids [71.0% (63.27–77.67%)] and in a model without extrinsic (year and site) or intrinsic (age and sex) factors, did not differ significantly (*χ*^2^_1_ = 0.88, *P* = 0.35). When fitted individually, none of the factors affected the prevalence of NSpir helminths in models with the P/A of spirurids. However, when all 4 factors were fitted concurrently, there was a weak significant effect of year on the prevalence of NSpir helminths in mice with and without spirurids (year × P/A spirurids × P/A NSpir helminths, *χ*^2^_3_ = 8.17, *P* = 0.043; Table S1). The prevalence of NSpir helminths was marginally higher in mice with spirurids, compared to those without spirurids in 2004 (87.0 *vs* 72.4%) and 2012 (74.7 *vs* 62.8%), but lower in 2000 (74.4 *vs* 83.3%) and 2008 (65.4 *vs* 71.4%).

A similar picture emerged when the analyses were confined to specific higher taxa of helminths, for example, the prevalence of NSpir nematodes was almost identical in mice with [63.7% (58.45–68.61%)] and without spirurids [64.9% (56.98–72.16%)]. Similarly, the prevalence of oxyuroid nematodes did not differ significantly between mice with or without spirurids [59.7% (54.41–64.77%) and 64.1% (56.21–71.44%), respectively; *χ*^2^_1_ = 0.18, *P* = 0.67, in a model with all 4 factors taken into consideration].

### Does the presence of spirurids affect prevalence of cestodes?

However, there was a marked, significant effect of the P/A of spirurids on the prevalence of cestodes (all cestode species combined; P/A spirurids × P/A cestodes, *χ*^2^_1_ = 9.90, *P* = 0.002), which retained independent significance when other factors were taken into account in the model (*χ*^2^_1_ = 6.01, *P* = 0.014). The prevalence of cestodes was 2.4 times higher in mice that additionally carried spirurid worms [20.0% (16.10–24.59%) and 8.4% (4.88–13.97%), respectively]. The bias in the prevalence of cestodes in favour of mice with spirurids was evident in most data subsets based on the levels within each of the 4 factors (Table 2; the only exception was in mice from Wadi Gharaba, where the prevalence of cestodes was markedly lower than that in other wadis).

**Table 2.** Prevalence of cestodes in spiny mice with or without spirurids by year, site, sex and host age class

		All cestodes						Adult, intestinal stages of cestodes			
		Spirurids present			Spirurids absent			Spirurids present		Spirurids absent	
Factor	Level	*n*	%	CL_95_	*n*	%	CL_95_	%	CL_95_	%	CL_95_
Year	2000	43	**16**.**3**	6.99–32.40	24	8.3	1.51–26.65	**9**.**3**	2.85–23.68	8.3	1.51–26.65
	2004	77	**19**.**5**	11.39–30.93	29	0	0.00–11.53	**14**.**3**	7.61–25.06	0	0–11.53
	2008	81	**21**.**0**	12.40–32.98	35	8.6	2.88–20.77	**17**.**3**	9.56–28.64	5.7	1.37–17.27
	2012	99	**21**.**2**	11.74–34.99	43	14.0	5.65–29.31	**21**.**2**	11.74–34.99	14.0	5.65–29.31
Site	El Arbaein	63	**31**.**7**	22.32–42.75	49	12.2	4.14–28.43	**30**.**2**	20.88–41.16	12.2	4.14–28.43
	Gebal	45	**26**.**7**	13.80–43.85	33	9.1	3.29–21.09	**22**.**2**	10.98–39.35	9.1	3.29–21.09
	Gharaba	98	3.1	0.46–12.11	14	**7**.**1**	0.37–31.71	0	0–7.34	0	0–23.81
	Tlah	94	**26**.**6**	16.21–39.98	35	2.9	0.32–13.16	**22**.**3**	12.88–35.69	2.9	0.32–13.16
Sex	Males	144	**13**.**2**	8.38–19.76	66	4.5	1.48–11.89	**10**.**4**	6.22–16.58	4.5	1.48–11.89
	Females	156	**26**.**3**	19.26–34.45	65	12.3	6.57–21.43	**22**.**4**	15.96–30.49	10.8	5.39–19.74
Age	Class 1	63	**7**.**9**	3.48–16.15	94	5.3	1.44–15.19	**7**.**9**	3.48–16.15	5.3	1.44–15.19
	Class 2	237	**23**.**2**	19.43–27.42	37	16.2	7.30–30.82	**19**.**0**	15.55–22.98	13.5	5.83–27.46

The higher prevalence at each level is highlighted in bold.

This relationship retained significance when the model was confined to intestinal (i.e. final) stages of cestodes (P/A spirurids × P/A intestinal stages of cestodes, *χ*^2^_1_ = 6.84, *P* = 0.009), and when the 4 factors were fitted in the model (*χ*^2^_1_ = 6.96, *P* = 0.008). The prevalence of intestinal stages of cestodes was 2.2 times higher in mice that additionally carried spirurid worms compared to those that were not infected with spirurids [16.7% (13.03–20.99% and 7.6% (4.33–13.02%), respectively; see Table 2 for breakdown of data by each of the 4 factors].

However, the relationship between P/A spirurids and the P/A of larval stages of cestodes was not significant, whether fitted alone (*χ*^2^_1_ = 3.55, *P* = 0.060) or when the 4 factors were taken into account (*χ*^2^_1_ = 0.171, *P* = 0.68).

### Does the presence of *P. muricola* affect prevalence of cestodes?

As *P. muricola* was the spirurid with the highest prevalence, we next focused on this species to assess whether it was the principal driver of the effect of spirurids on the prevalence of cestodes. The prevalence of cestodes was indeed higher in mice with *P. muricola* [19.3% (15.89–23.24) *vs* 13.3 (10.54–16.62)] but this was just the wrong side of the cut-off for significance (*χ*^2^_1_ = 2.84, *P* = 0.092). However, in a model with all 4 factors, the interaction between P/A *P. muricola* and P/A cestodes was site dependent (*χ*^2^_3_ = 8.67, *P* = 0.034). The bias in favour of mice with spirurids was clearly evident among the animals from Wadi El Arbaein [33.3% (24.22–43.86) *vs* 12.7% (7.04–21.06)] and from Wadi Tlah [28.4% (18.33–40.73) *vs* 6.3% (1.15–20.49)], which mainly accounted for this site effect. In Wadi Gharaba where *P. muricola* was most prevalent (79.5%), cestodes were rare (3.6%), whereas in Wadi Gebal, only 1 of the 78 mice sampled from this location was infected with *P. muricola* (prevalence *=* 1.3%).

Therefore, as *P. muricola* was so rare in Wadi Gebal, we repeated the analysis confining it to mice from Wadis El Arbaein, Gharaba and Tlah. The prevalence of cestodes in mice with *P. muricola* was now higher relative to mice without *P. muricola* [19.4% (15.97–23.32) *vs* 9.5 (5.79–15.09)] and significant (*χ*^2^_1_ = 6.31, *P* = 0.012), and when the 4 factors were taken into account, this was still a significant term, although site dependent, as found earlier (P/A *P. muricola* × P/A all cestodes × site, *χ*^2^_2_ = 8.013, *P* = 0.018). The site effect arose because, although in Wadis El Arbaein [33.3% (24.22–43.86) *vs* 12.7% (7.04–21.06)] and Tlah [28.4% (18.33–40.73) *vs* 6.3% (1.15–20.49)] prevalence of cestodes was higher among mice with *P. muricola*, in Wadi Gharaba where cestodes were relatively rare, prevalence was 2.2% (0.25–10.26) among mice with *P. muricola*, but higher in those without *P. muricola* 8.7% (1.57–27.81).

The prevalence of intestinal stages of cestodes was 16.7% (13.43–20.43) in mice with *P. muricola* but lower at 10.8% (8.32–13.91) in mice that were not infected with *P. muricola* (*χ*^2^_1_ = 6.412, *P* = 0.011), and independent of the 4 factors. The difference was even more marked without mice from Wadi Gebal [16.7% (13.50–20.50) *vs* 7.1% (4.04–12.25), respectively] and highly significant (P/A *P. muricola* × P/A intestinal cestodes, *χ*^2^_1_ = 7.280, *P* = 0.007).

### Does the presence of spirurids affect prevalence of acanthocephalans?

The acanthocephalan, *Moniliformis acomysi* Ward and Nelson, 1967, was found only in mice with spirurid worms, in which prevalence of the acanthocephalan was 8.0% (5.57–11.41). In a model with all 4 factors this was a highly significant effect (P/A spirurids × P/A *M. acomysi*, *χ*^2^_1_ = 18.003, *P* < 0.001), and statistically independent of the 4 factors. Nevertheless, there was some variation in the prevalence of *M. acomysi* in mice with spirurid worms. The highest prevalence in mice concurrently carrying spirurids was in Wadi Gharaba [18.4% (9.76–31.25)], whereas in Wadi Gebal, none of the mice with spirurids (*n* = 45) were infected with this acanthocephalan [0.0% (0.00–10.24)].

As *M. acomysi* was absent from the mice in Wadi Gebal, and the prevalence of *P. muricola* was extremely low (1.3%), we fitted a model without the data from this wadi. The outcome was much the same (P/A *P. muricola* × P/A *M. aco*mysi, *χ*^2^_1_ = 17.043, *P* < 0.001, and independent of the 4 factors). The prevalence of *M. acomysi* in mice with *P. muricola* was 10.6% (7.98–13.78) and 0.0 (0–2.36) in mice without *P. muricola*.

### Does the presence of spirurids or *P. muricola* affect species richness with non-spirurid helminths?

Although mean NSpir helminth species richness was numerically higher in mice with spirurids, compared with those that were not infected with spirurids (1.23 ± 0.058 and 1.02 ± 0.075, respectively), this difference was not significant in a GLM with Poisson errors (Dev_1,429_ = 3.542, *P* = 0.06), nor in an MSM with all significant factors (year, site and sex) taken into account (Dev_1,422_ = 3.303, *P* = 0.07; Table S2). However, with the exception of mice from Wadi Gharaba and those sampled in 2000, mean NSpir helminth species richness was numerically higher in all the remaining data subsets (Table S2).

The mean number of NSpir helminth species also did not differ significantly according to whether mice were or were not infected with *P. muricola* (Table S3; 1.25 ± 0.066 and 1.06 ± 0.078, respectively; Dev_1,351_ = 2.554, *P* = 0.11; analysis excluding mice from Wadi Gebal), nor when all significant factors (year, site, age and sex), and their interactions, had been taken into account (Dev_1,345_ = 2.050, *P* = 0.15).

### Does the presence of spirurids affect abundance of other helminths?

The summary data in Table 3 show that for the listed taxa, mean worm burdens were numerically higher in mice with spirurid worms compared to those without spirurids in all cases, although these values do not take into account differences in abundance between the wadis, years, sexes and age classes. However, with all significant terms, including interactions between extrinsic and intrinsic factors taken into account, the spirurid-infected mice had significantly higher worm burdens of NSpir helminths (GLM with negbin errors, LR_1,390_ = 4.780, *P* = 0.029). Mean worm burdens were higher in spirurid-infected mice in each year, in each site, both sexes and both age classes (Table 4). For NSpir nematodes and for oxyuroid nematodes the differences between mice with/without spirurids were not significant (LR_1,399_ = 2.911, *P* = 0.088 and LR_1,402_ = 3.175, *P* = 0.075, respectively), although close to the cut-off for significance. GLMs with negative binomial error structures for all cestodes, larval cestodes and acanthocephalans would not converge, but that for the intestinal stages of cestodes did, and revealed a highly significant difference for worm burdens of mice with/without spirurids (LR_1,411_ = 12.527, *P* = 0.0004; Table 5).

**Table 3.** Mean worm burdens of non-spirurid (NSpir) helminth taxa in spiny mice infected and uninfected with spirurid nematodes

	Spirurids present (*n* = 300)		Spirurids absent (*n* = 131)		*P. muricola* present (*n* = 227)		*P. muricola* absent (*n* = 126)	
	Mean ±	s.e.m.	Mean ±	s.e.m.	Mean ±	s.e.m.	Mean ±	s.e.m.
NSpir helminths	**32**.**3**	9.96	10.6	3.14	**38**.**2**	13.09	13.8	3.70
NSpir nematodes	**28**.**8**	9.94	10.2	3.12	**34**.**9**	13.09	11.5	3.44
Oxyuroids	**27**.**4**	9.92	10.1	3.12	**33**.**1**	13.07	11.3	3.44
All cestodes	**2**.**22**	0.733	0.43	0.308	1.6	0.53	**2**.**3**	1.48
Intestinal adult stages	**0**.**54**	0.112	0.12	0.047	**0**.**52**	0.118	0.15	0.062
Larval stages	**1**.**68**	0.729	0.31	0.305	1.06	0.525	**2**.**17**	1.483
Acanthocephala	**1**.**290**	0.585	0	0	**1**.**70**	0.771	0	0

The higher abundance is highlighted in bold.

These values do not take into account differences between wadis, years, sexes and age classes. Values in the columns for *P. muricola* present and absent are confined to mice from Wadis El Arbaein, Gharaba and Tlah, omitting Wadi Gebal because *P. muricola* was extremely rare in the latter wadi (see Behnke *et al*., 2019).

**Table 4.** Abundance of NSpir helminths in spiny mice with/without spirurids, and with/without *P. muricola* by year, site, host sex and age class

		Spirurids present			Spirurids absent			*P. muricola* present			*P. muricola* absent		
Factor	Level	*n*	Mean ±	s.e.m.	*n*	Mean ±	s.e.m.	*n*	Mean ±	s.e.m.	*n*	Mean ±	s.e.m.
Year	2000	43	**10.56**	2.953	24	8.83	2.991	32	8.00	1.756	21	**14.29**	5.86
	2004	77	**63**.**14**	36.336	29	22.97	13.205	59	**77**.**85**	47.298	26	29.08	15.242
	2008	81	**19**.**51**	5.437	35	6.46	2.054	58	**19**.**17**	6.888	38	9.711	4.673
	2012	99	**28**.**30**	9.372	43	6.74	2.407	78	**34**.**76**	11.795	41	7.59	2.591
Site	El Arbaein	63	**17**.**60**	6.124	49	3.37	0.689	57	**17**.**12**	6.447	55	5.42	2.372
	Gebal	45	**10**.**04**	2.652	33	7.03	2.200	—	—	—	—	—	—
	Gharaba	98	**16**.**86**	4.385	14	7.29	3.811	89	**16**.**44**	4.471	23	12.65	7.66
	Tlah	94	**68**.**99**	30.934	35	25.57	11.149	81	**76**.**95**	35.825	48	23.90	8.428
Sex	Males	144	**16**.**97**	3.757	66	10.17	5.269	102	**18**.**00**	4.976	69	13.26	5.522
	Females	156	**46**.**51**	18.792	65	11.12	3.419	125	**54**.**69**	23.368	57	14.40	4.760
Age	Class 1	63	**13**.**57**	4.400	94	13.49	4.305	48	**16**.**73**	5.704	82	13.59	4.876
	Class 2	237	**37**.**31**	12.537	37	3.41	1.516	179	**43**.**96**	16.518	44	14.14	5.513

The higher mean worm burden at each level is highlighted in bold.

Data from Wadi Gebal were excluded for columns under P/A *P. muricola*, because this species was extremely rare in this wadi.

**Table 5.** Abundance of cestodes in spiny mice with or without spirurids by year, site, host sex and age class

		All cestodes						Adult, intestinal stages of cestodes			
		Spirurids present			Spirurids absent			Spirurids present		Spirurids absent	
Factor	Level	*n*	Mean	s.e.m.	*n*	Mean	s.e.m.	Mean	s.e.m.	Mean	s.e.m.
Year	2000	43	**2**.**16**	1.422	24	0.13	0.092	**0**.**21**	0.127	0.13	0.092
	2004	77	**2**.**21**	1.234	29	0	0	**0**.**16**	0.046	0	0
	2008	81	**4**.**11**	2.313	35	1.20	1.142	**0**.**88**	0.325	0.06	0.040
	2012	99	**0**.**71**	0.195	43	0.26	0.129	**0**.**71**	0.195	0.26	0.129
Site	El Arbaein	63	**1**.**22**	0.350	49	0.22	0.114	**0**.**94**	0.294	0.22	0.114
	Gebal	45	**1**.**47**	0.619	33	0.12	0.072	**0**.**82**	0.438	0.12	0.072
	Gharaba	98	**3**.**18**	2.015	14	2.86	2.857	0	0	0	0
	Tlah	94	**2**.**24**	0.969	35	0.03	0.029	**0**.**70**	0.203	0.03	0.029
Sex	Males	144	**1**.**28**	0.639	66	0.05	0.026	**0**.**19**	0.057	0.05	0.026
	Females	156	**3**.**08**	1.279	65	0.82	0.619	**0**.**87**	0.206	0.20	0.091
Age	Class 1	63	**0**.**29**	0.165	94	0.11	0.059	**0**.**29**	0.165	0.11	0.059
	Class 2	237	**2**.**73**	0.925	37	1.24	1.079	**0**.**61**	0.134	0.16	0.073

The higher mean worm burden at each level is highlighted in bold.

Sample sizes (*n*) for adult intestinal stages of cestodes are the same as those for all cestodes.

When the P/A of *P. muricola* was fitted as the explanatory factor, and analysis was confined to records from Wadis El Arbaein, Gharaba and Tlah, omitting Wadi Gebal, because of the rarity of *P. muricola* in this site, there was no significant effect on NSpir helminths (LR_1,326_ = 3.079, *P* = 0.079), or NSpir nematodes (LR_1,323_ = 3.009, *P* = 0.083). Nevertheless, in both cases *P* was close to the cut-off for significance and as the data in Table 4 show, mean worm burdens were numerically higher in *P. muricola*-infected mice in 3 of the 4 surveys (the exception was in 2000), in all 3 sites, both sexes and both age classes. However, oxyuroid nematode worm burdens were significantly higher in mice with *P. muricola* (LR_1,304_ = 4.229, *P* = 0.039; Table 3) as were also those of intestinal cestodes (LR_1,334_ = 7.639, *P* = 0.0057; Table 3).

Acanthocephala were found only in mice concurrently infected with spirurid worms, and particularly with *P. muricola*.

### Does the increasing intensity of combined spirurid or *P. muricola* worm burdens covary with increasing species richness of non-spirurid (NSpir) helminths?

The mean values for NSpir helminth species richness increased with increasing mean spirurid worm burdens as reflected in spirurid intensity classes, based on raw data (Table 6). To control for the possible confounding effects of intrinsic and extrinsic factors known to affect helminth abundance, the association was tested by inclusion of the residuals of the MSM for the combined spirurid worm burdens as a covariate in a model with the MSM for NSpir helminth species richness. The residuals of the MSM for the combined spirurid worm burden were a highly significant explanatory term in this model with a positive gradient, significantly different to zero (Table 7).

**Table 6.** Worm burdens of non-spirurid helminth taxa in mice with increasing worm burdens of spirurid worms, aggregated in intensity classes

Class	1	2	3	4	5	6
*n*	131	105	75	47	53	20
Range	0	1–4	5–9	10–19	20–49	>49
Mean	0	2.3	6.9	13.0	33.7	95.6
s.e.m.	0	0.11	0.16	0.39	1.20	10.82
Mean worm burden ± s.e.m.						
All other helminths	10.6 ± 3.14	13.0 ± 3.21	17.3 ± 5.05	98.0 ± 60.70	28.2 ± 10.14	46.4 ± 18.25
All other nematodes	10.2 ± 3.12	10.4 ± 2.78	15.0 ± 5.03	94.8 ± 60.75	24.9 ± 10.10	32.5 ± 17.64
Oxyuroidea	10.1 ± 3.12	10.2 ± 2.78	14.4 ± 5.02	87.8 ± 60.80	24.2 ± 10.12	32.1 ± 17.67
All cestodes	0.43 ± 0.308	2.57 ± 1.741	1.73 ± 0.944	1.15 ± 0.532	1.85 ± 1.267	5.70 ± 3.553
Intestinal cestodes	0.12 ± 0.047	0.34 ± 0.114	0.41 ± 0.138	0.64 ± 0.411	0.60 ± 0.288	1.65 ± 0.812
Larval cestodes	0.31 ± 0.305	2.23 ± 1.742	1.32 ± 0.940	0.51 ± 0.358	1.25 ± 1.245	4.05 ± 3.559
Acanthocephala	0 ± 0	0.05 ± 0.048	0.57 ± 0.395	2.11 ± 2.063	1.43 ± 0.636	8.20 ± 6.935
Mean species richness^a^	1.02 ± 0.075	1.14 ± 0.103	1.21 ± 0.106	1.26 ± 0.141	1.28 ± 0.125	1.60 ± 0.285

Note that these summary stats are for means of raw data, without taking into account any variation arising from year, site, sex or age.

a This is helminth species richness excepting the Spirurida.

**Table 7.** Quantitative covariance of species richness and worm burdens of non-spirurid (NSpir) helminth taxa with worm burdens of the Spirurida (SpirWB) or with those of *P. muricola* (PmWB)

Taxon	Model^a^	Statistics and value^b^		*P*^b^	Slope (*β*)	± s.e.m.	*z*^c^	*P*^c^
Species richness	Poisson MSM + SpirWB	Dev_1,422_	8.254	0.0041	0.1343	0.04634	2.898	0.00375
Species richness	Poisson MSM + PmWB	Dev_1,345_	8.443	0.0037	0.1536	0.05223	2.940	0.00328
NSpir helminths	Negbin^d^, MSM + SpirWB	LR_1,390_	10.283	0.0013	0.3208	0.08882	3.612	0.00030
NSpir helminths	Negbin, MSM + PmWB	LR_1,326_	7.785	0.0053	0.3282	0.10325	3.179	0.00148
NSpir nematodes	Negbin MSM + SpirWB	LR_1,390_	10.200	0.0014	0.3659	0.10171	3.597	0.00032
NSpir nematodes	Negbin MSM + PmWB	LR_1,326_	9.150	0.00248	0.4152	0.11724	3.542	0.00040
Oxyuroidea	Negbin MSM + SpirWB	LR_1,402_	10.382	0.00127	0.3748	0.10534	3.558	0.00037
Oxyuroidea	Negbin MSM + PmWB	LR_1,328_	8.902	0.00285	0.4102	0.12250	3.349	0.00081
Intestinal cestodes	Negbin MSM + SpirWB	LR_1,411_	8.853	0.00293	0.5203	0.16340	3.185	0.00145
Intestinal cestodes	Negbin MSM + PmWB	LR_1,334_	4.076	0.04349	0.4051	0.19620	2.064	0.03900

Models comprised of the residuals of the MSMs for combined SpirWB or PmWB as covariates fitted as additional explanatory variables to MSMs of the specified taxa.

a In each case we first derived MSMs for the specified taxon from full factorial models (year × site × age × sex) and then added the residuals of MSMs for SpirWB or PmWB as covariates. Models that included PmWB as a covariate were confined to data from Wadis El Arbaein, Gharaba and Tlah, because *P. muricola* was extremely rare among mice from Wadi Gebal (see text for more details and Behnke *et al*., 2019).

b Test statistics (Dev, deviance for models with Poisson errors; LR, likelihood ratio for models with negative binomial errors, both distributed as chi-squared) with degrees of freedom, its value and *P* for comparison of MSMs with and without SpirWB or PmWB (covariates).

c *z* and *P* value for test of whether the slope differs from a slope of zero.

d Model with negative binomial error structure.

Models with *P. muricola* worm burdens as the explanatory factor were restricted to the 3 sites in which this species was encountered. As above, we fitted the residuals of the MSM for *P. muricola* worm burdens as a covariate, in a model with the MSM of NSpir helminth species richness. With site, year, sex, age and all significant interactions taken into account, worm burdens of *P. muricola* were a significant explanatory term in this model with a positive gradient that was significantly different to zero (Table 7).

**Figure 2.**
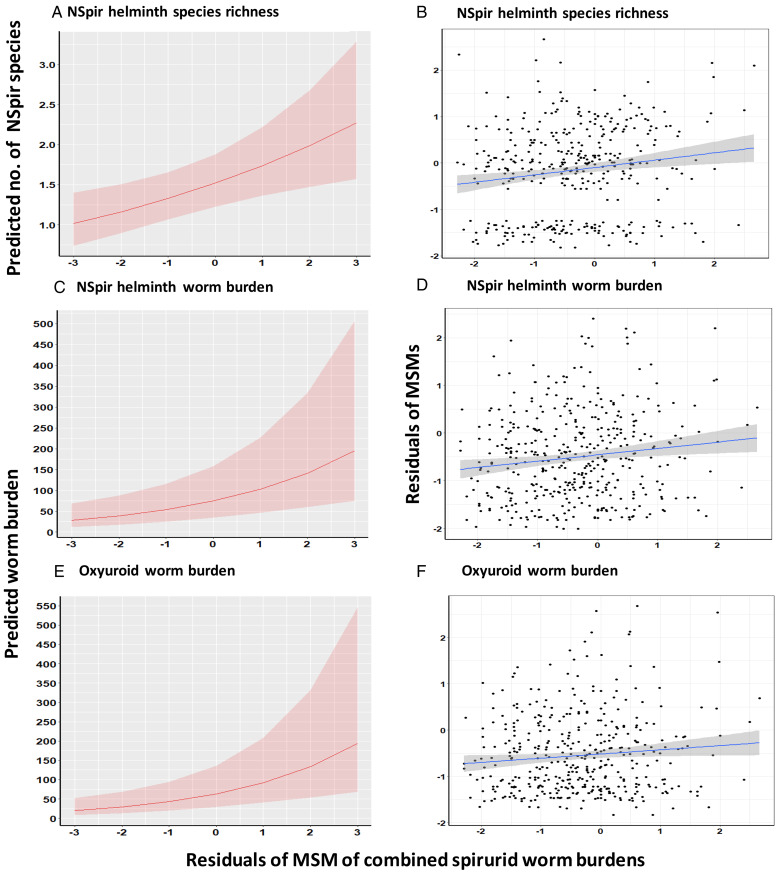
Covariance of the residuals of the MSMs for the combined spirurid worm burdens with NSpir helminth species richness (A and B), NSpir helminth worm burdens (C and D) and oxyuroid worm burdens (E and F). The panels on the left (A, C and E) illustrate the predictions of the relevant models in Table 7; those on the right (B, D and F) show regressions of MSMs of NSpir helminth species richness (B; *R*^2^ = 0.0234), NSpir helminth (D; *R*^2^ = 0.0178) and oxyuroid (F; *R*^2^ = 0.0104) worm burdens on the MSM for the combined spirurid worm burdens. The shaded areas show the 95% confidence region. For additional details, see text and Tables 7 and S4.

In an alternative approach, we also tested the relationships between the residuals of the MSMs for NSpir helminth species richness and those of combined spirurid or *P. muricola* worm burdens and both were highly significant (Table S4, but see also legend to Fig. 2), although these accounted for just 2.3 and 3.1% of the variance, respectively.

### Does the increasing intensity of combined spirurid or *P. muricola* worm burdens covary with increasing intensities of non-spirurid (NSpir) helminth taxa?

The same approach was implemented to assess the degree of covariance of combined spirurid and *P. muricola* worm burdens on combined NSpir helminth, NSpir nematode, oxyuroid nematode and intestinal cestode worm burdens. In all cases, these relationships were significant with positive slopes (Tables 7 and S4, and Fig. 2A–F), but in linear models testing covariance between the MSM of these taxa (Table S4) none explained more than 1.9% of the variance.

As the records contained multiple cases of uninfected animals with relevant taxa, and because concentrations of double negatives can generate false-positive slopes in models of covariance, we repeated the analysis of covariance of residuals from MSMs of relevant taxa, but this time selecting only mice that had at least 1 worm of each taxon. These relationships are illustrated in Figs S1 and S2, with statistical analyses provided in the figure legends. With the exception of models that included intestinal cestodes, all revealed positive slopes that were either significant or close to significance, but none explained more than 3.1% of variance.

In an alternative approach, we used the covariance distribution test of Haukisalmi and Henttonen (1998), focusing first on covariance of combined spirurid worm burdens *vs* NSpir helminths (cov = 0.404, *P* < 0.00001), NSpir nematodes (cov = 0.183, *P* = 0.034), oxyuroid nematodes (cov = 0.123, *P* = 0.102) and intestinal cestodes (cov = 0.102, *P* = 0.0004). All these covariances were positive and retained, or even gained significance when subset grouping (the 4 factors) was taken into account (*P* < 0.00001, *P* = 0.0046, *P* = 0.0090 and *P* < 0.00001, respectively). The test was next applied to *P. muricola* worm burdens (excluding data from Wadi Gebal) *vs* NSpir helminths (cov = 0.436, *P* = 0.0002), NSpir nematodes (cov = 0.204, *P* = 0.04320), oxyuroids (cov = 0.134, *P* = 0.1298) and intestinal cestodes (cov = 0.103, *P* = 0.0026). Again, all these covariances were positive and retained, or even gained significance when subset grouping (the 4 factors) was taken into account (*P* = 0.0002, *P* = 0.0030, *P* = 0.0032 and *P* = 0.0022, respectively).

### Does the biomass of spirurid worm burdens affect abundance of other helminths?

**Figure 3.**
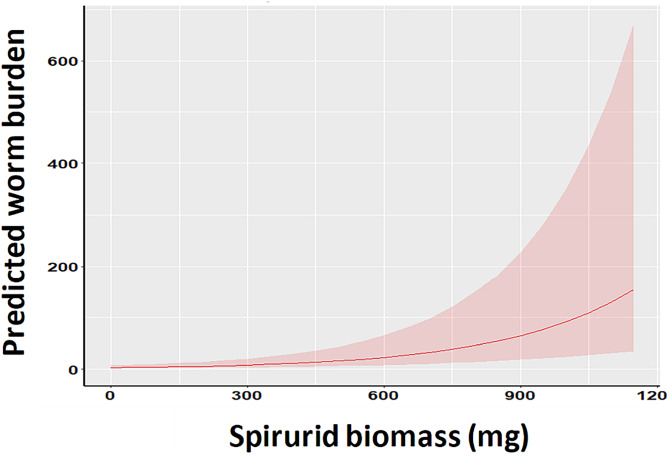
Covariance of the residuals of the MSMs for the combined oxyuroid worm burdens with spirurid biomass.

Spirurid biomass was available for 228 mice that carried either *P. muricola* or *M. muris* or both. MSMs were fitted for species richness of NSpir helminths, NSpir helminth worm burdens, oxyuroid nematodes and intestinal cestodes in these mice, and the 131 mice that were not infected with either of these species (total *n* = 359). These were then compared to MSMs with spirurid biomass, the latter term fitted as a covariate. The results are summarized in Table 8. With all extrinsic and intrinsic factors taken into account, the only covariance that was significant was that for oxyuroid nematodes *vs* spirurid biomass (Table 8 and Fig. 3). The slope for this relationship was positive and highly significant. The slope for NSpir helminths was also positive and significantly different from zero, but the difference between the MSM with and without spirurid biomass was not.

**Table 8.** Quantitative covariance of species richness and worm burdens of non-spirurid helminth taxa with the biomass of Spirurida comprising *P. muricola*, *M. muris* or both species

Taxon	Model^a^	Statistics and value^b^		*P*^b^	Slope (*β*)	± s.e.m.	*z*^c^	*P*^c^
Species richness	Poisson	Dev_1,352_	0.910	0.3402	0.000261	0.00027	0.969	0.3325
NSpir helminths	Negbin^d^	LR_1,320_	2.924	0.0873	0.00118	0.000517	2.275	0.0229
Oxyuroidea	Negbin	LR_1,327_	13.234	0.00027	0.00347	0.000605	5.736	<0.00001
Intestinal cestodes	Negbin	LR_1,345_	0.890	0.3455	0.00108	0.00122	0.881	0.3785

a In each case we first derived MSMs for the specified taxon from full factorial models (year × site × age × sex) and then added spirurid biomass as a covariate.

b Test statistics (Dev, deviance for models with Poisson errors; LR, likelihood ratio for models with negative binomial errors, both distributed as chi-squared) with degrees of freedom, its value and *P* for comparison of MSMs with and without spirurid biomass (covariate).

c *z* and *P* value for test of whether the slope differs from a slope of zero.

d Model with negative binomial error structure.

## Discussion

Our data clearly show that infection with spirurid nematodes has significant effects on the likelihood of infection with other helminth taxa and that to some extent this relationship is intensity-dependent. Initially, we found that the prevalence of non-spirurid helminths, non-spirurid nematodes and the Oxyuroidea did not differ between mice harbouring spirurid nematodes, or the dominant species *P. muricola*, and those not infected with parasites of these taxa. However, the likelihood of infection with cestodes, but especially intestinal stages and the acathocephalan *M. acomysi*, was significantly higher. Not only was the prevalence of cestodes and the intestinal stages of cestodes higher overall in spirurid-carrying mice, but this bias for higher cestode prevalence was evident in all 4 surveys, in both sexes, both age classes and in 3 of the 4 sites where we trapped rodents.

Spirurid nematodes have indirect life cycles with coprophagous arthropod hosts playing an essential role as intermediate hosts (Chabaud, 1954). Species of *Gongylonema* have been shown to be transmitted by dung beetles (Scarabaeidae; Mowlavi *et al*., 2009; Mukaratirwa *et al*., 2010). The intermediate hosts of *P. muricola* and *M. muris* include earwigs (Dermaptera), cockroaches (Blattodea), fleas (Siphonaptera) and tenebrionid beetles (Coleoptera) (Quentin, 1969, 1970; Shogaki *et al*., 1972; Campos and Vargas, 1977; Schutgens *et al*., 2015) and those of *Streptopharagus* spp., tenebrionid beetles (Chabaud, 1954; Wertheim, 1993; Montoliu *et al*., 2013). Intermediate arthropod hosts are also exploited by cestodes (Wardle and McLeod, 1952) and by acanthocephalans (Moore, 1946; Crompton and Nickol, 1985; Moore and Crompton, 1993), and although the exact arthropod species may vary between helminth taxa, cockroaches have been shown to act as intermediate hosts for both spirurids and acanthocephalans (Quentin, 1969, 1970; Shogaki *et al*., 1972; Moore and Crompton, 1993). Representative species of all these arthropod taxa are known to exist in the wadis in the Egyptian Sinai, and especially in and around the Bedouin gardens where our trapping was concentrated (Semida *et al*., 2001; Zalat *et al*., 2001; Norfolk *et al*., 2012). *Acomys* spp. are known to be omnivorous, feeding on arthropods, green vegetation, seeds and snails, depending on season and local availability of these food categories, but with a dietary preference for arthropods (Kronfeld-Schor and Dayan, 1999). It is likely therefore, that to some extent our results can be explained by the availability of intermediate hosts, carrying the larval stages of spirurid nematodes, cestodes and the acanthocephalan, as a food resource for the spiny mice. In this context, it is interesting that *M. acomysi* was only found in mice that were concurrently infected with spirurids, suggesting strongly that the mice fed on arthropods infected with the intermediate stages of both taxa, and the most likely invertebrate hosts were the locally common cockroaches (Hassan and Fadl, 2000; Zalat *et al*., 2008; J.M. Behnke *et al*., unpublished observations).

Species richness of non-spirurid taxa was not affected significantly by the presence of spirurid worms, although the mean values for species richness of non-spirurid taxa were all numerically higher for mice harbouring spirurids, and while not significant, the statistical models were highly suggestive of a trend in this direction, perhaps suggesting a weak effect. We also found that mice carrying spirurids had higher worm burdens of NSpir helminths, compared to those without spirurids. This effect was very prominent in each year of the study, in each site, in both sexes and in both age classes. Not unexpectedly given this outcome of the analysis, there was a significant quantitative relationship, reflected in covariance between the intensity of infection with spirurids and NSpir helminth species richness, as well as with NSpir helminth worm burdens, but these were relatively weak effects accounting for only 2.3 and 3.1% of the variance, respectively.

The mean abundances of NSpir nematodes and oxyuroids were both numerically higher in mice with spirurids or with *P. muricola*, compared to mice without these taxa, but statistical tests only generated *P* values of between 0.05 and 0.1, again highly suggestive of an effect but not conclusive. Significant quantitative covariance of these taxa with spirurid worm burdens however indicated that higher worm burdens with spirurid nematodes or with *P. muricola* were associated with higher worm burdens of NSpir nematodes and oxyuroid nematodes. Although such results can also be explained to some extent by reliance on a diet of arthropod intermediate hosts in the case of NSpir helminths, this cannot be the explanation for the oxyuroid nematodes, which are all directly transmitted, without involvement of intermediate hosts.

An obvious explanation for higher worm burdens of NSpir taxa in spirurid-infected mice may lie in exacerbated host weakness arising from pathology generated by heavy spirurid worm burdens. Although our surveys only revealed a single *Pterygodermatites witenbergi* Quentin and Wertheim, 1975, species of *Pterygodermatites* are large worms and their presence has been linked to local inflammation and damage to the intestine. Moreover, *Pterygodermatites peromysci* have been suggested to destabilize populations of its rodent host *Peromyscus leucopus* (Vandegrift and Hudson, 2009). *Gongylonema* spp. burrow through the oeosophageal and gastric mucosa, generating marked pathology as they do so, reflected in fibrinous, eosinophilic inflammation, distortion and disruption of the mucosal lining (Chakraborty, 1994; Kheirandish *et al*., 2013). Human case reports of infection with *Gongylonema* spp. document nausea, persistent vomiting and unpleasant oral symptoms (Libertin *et al*., 2017; Xiaodan *et al*., 2018). *Protospirura muricola* has been shown to reduce both stomach and host weight in heavily infected laboratory mice, and to cause marked pathology in monkeys, resulting in the death of these hosts in some cases (Foster and Johnson, 1939; Lowrie *et al*., 2004; Navarro-Serra *et al*., 2021). In our study, some spiny mice had such heavy infections with *P. muricola*, that the stomach walls bulged outwards, and on dissection revealed large knots of worms occupying most of the lumen (Fig. 1), and much the same was observed in mice infected with *M. muris* (but see Krauss, 1977, for pathology of this species in common voles, *Microtus arvalis*).

Alternatively, helminths that cause long-lasting chronic infections have been shown to facilitate their own survival by the secretion of agents that interfere with and impair the normal function of mucosal immune responses (Behnke *et al*., 2009*a*; Hewitson *et al*., 2011; Varyani *et al*., 2017; Bancroft and Grencis, 2021), as well as affecting physiological processes in the intestinal tract (Hayes and Grencis, 2021; Lane *et al*., 2023). Although, nothing is currently known about such mechanisms among the Spirurida, *P. muricola* for example has been shown to survive for many weeks after infection of laboratory mice. An age-dependent increase in the abundance of these worms in naturally infected *A. dimidiatus* (Behnke *et al*., 2019) implies that this species also exploits some, as yet unknown, mechanism to impair host defences and favour its own survival. If spirurids turn out to facilitate their own survival in mammalian hosts by downregulating local immune responses in the stomach, these may also have downstream consequences in more posterior regions of the intestine and enable other concurrently residing helminths, such as the oxyuroid nematodes in this study, to benefit from weakened mucosal immunity (Behnke *et al*., 1978, 2001).

Field-based cross-sectional studies of interactions between parasites in concurrently infected hosts, based on culled hosts, have been criticized in recent years and alternative approaches have been advocated involving repeated assessment of marked individuals through fecal egg/cyst counts conducted over time (Knowles *et al*., 2013; Fenton *et al*., 2010, 2014; Sweeny *et al*., 2021; but see also Haukisalmi and Henttonen, 1998; Barger, 2023, for evaluation of alternative statistical models). Nonetheless, this is arguably still an unresolved area: incontestable, known interaction backgrounds against which to benchmark methods in wild systems are difficult to contemplate and system-specific considerations may apply, including the reliability of measurement (Fenton *et al*., 2014; Barger, 2023). Moreover, interactions would indeed be expected to be detectable in cross-sectional data under some conditions, and to return the correct interaction sign sometimes (Bottomley *et al*., 2005). Thus, despite the important sources of uncertainty that arise from unmeasured heterogeneities in cross-sectional studies, stronger emphasis might be placed on cross-sectional data where confounder-adjusted relationships between parasite taxa can be repeatedly demonstrated in replicated localities and successive surveys conducted over a long period of time, as in the decade-long current study (but see also Behnke *et al*., 2001, 2009*b*, 2019, 2021; Lewis *et al*., 2023). In such cases, the observed trends are more likely to result from general processes, including competition or facilitation, rather than local idiosyncrasies of transmission, specific to a particular time and place. Interaction may also be more strongly indicated where the candidate interactors have obviously different transmission processes, such as in the case of Oxyuroidea *vs* the trophically transmitted helminths here.

In this study, we have shown that the presence of spirurid worms in spiny mice has marked consequences for the likelihood of being infected with other helminth taxa. We conclude that although the prevalence of other nematode taxa did not differ between spirurid-infected and uninfected mice, those of the intestinal stages of cestodes and acanthocephalans did. Moreover, the worm burdens of other helminths were heavier in spirurid-infected mice and marginally dose-related with concurrently present spirurid burdens. Our study was conducted only in the late summer period, but in the same months of each of the 4 survey years. The dietary preferences of spiny mice vary throughout the year, depending on availability of resources, and while they are thought to breed throughout most of the year, breeding is most intense during local rainy periods (Kronfeld-Schor and Dayan, 1999; Sari *et al*., 2016). Transmission of helminths is therefore also likely to vary between seasons, and the associations that we detected in the current study in summer months may differ at other times of the year. Although this is a limitation of the current study, possible temporal variation is clearly a topic for further research. Nevertheless, based on our data, acquired in the late summer months, we propose that the co-occurrence of spirurids with the arthropod-transmitted NSpir helminths is most parsimoniously explained by the hosts' reliance on a diet of arthropods, which happen locally to serve as intermediate hosts of these taxa. However, given that heavy infections with the stomach dwelling spirurid worms have adverse consequences for intestinal homoeostasis, our results imply that the resultant weakened mucosal immunity may favour the survival of other helminth species to which the mice are exposed. These include the Oxyuroidea that have direct life cycles and are not dependent on arthropods, as intermediate or paratenic hosts, to complete their life cycles.

## Supporting information

Behnke et al. supplementary material Behnke et al. supplementary material

## Data Availability

The data employed in the analyses reported in this paper can be made available from the corresponding author on reasonable request.

## Supplementary material

The supplementary material for this article can be found at https://doi.org/10.1017/S0031182024000891.
