# Supplementary material for: Large-bodied gastric spirurids (Nematoda, Spirurida) predict structure in the downstream gastrointestinal helminth community of wild spiny mice (Acomys dimidiatus)
Source: Parasitology. 2024 Sep 25;151(8):808–20. doi: 10.1017/S0031182024000891 (PMC11579037; doi:10.1017/S0031182024000891)
Supplement: Behnke et al. supplementary material [file S0031182024000891sup001.docx]

**Supplementary files**

**Table S1.** Prevalence of NSpir helminths (i.e., not Spirurida) in spiny mice

with or without spirurids by year, site, sex, and host age class.

__________________________________________________________________

Spirurids present Spirurids absent

____________________ ______________________

Factor Level *n* % CL_95_ *n* % CL_95_

___________________________________________________________________

Year 2000 43 74.4 57.68-86.59 24 **83.3** 62.76-94.09

2004 77 **87.0** 76.73-93.40 29 72.4 53.50-86.39

2008 81 65.4 52.97-76.40 35 **71.4** 56.40-83.29

2012 99 **74.7** 60.92-85.09 43 62.8 45.93-77.21

Site El Arbaein 63 **69.8** 58.84-79.12 49 65.3 47.16-80.63

Gebal 45 **75.6** 58.40-87.62 33 63.6 48.98-76.35

Gharaba 98 69.4 55.59-80.68 14 **71.4** 42.57-89.59

Tlah 94 85.1 72.82-92.72 35 **85.7** 71.89-93.55

Sex Males 144 **72.2** 64.27-79.06 66 69.7 58.46-79.15

Females 156 **78.2** 70.25-84.52 65 72.3 61.46-81.33

Age Class 1 63 **79.4** 69.15-87.09 94 76.6 63.24-86.40

Class 2 237 **74.3** 69.91-78.20 37 56.8 41.16-71.24

Combined 300 **75.3** 70.52-79.59 131 71.0 63.27-77.67

___________________________________________________________________

The higher prevalence at each level is highlighted in bold.

**Table S2.** NSpir helminth species richness (i.e., not Spirurida) in spiny mice

with or without spirurids by year, site, sex, and host age class.

_________________________________________________________________

Spirurids present Spirurids absent

_______________ _________________

Factor Level *n* Mean SEM *n* Mean SEM

_________________________________________________________

Year 2000 43 1.09 0.140 24 **1.17** 0.167

2004 77 **1.64** 0.109 29 1.10 0.167

2008 81 **1.04** 0.120 35 1.00 0.153

2012 99 **1.14** 0.093 43 0.91 0.128

Site El Arbaein 63 **1.21** 0.136 49 0.90 0.118

Gebal 45 **1.36** 0.159 33 0.82 0.134

Gharaba 98 0.98 0.083 14 **1.07** 0.286

Tlah 94 **1.46** 0.107 35 1.37 0.143

Sex Males 144 **1.15** 0.082 66 0.89 0.089

Females 156 **1.31** 0.081 65 1.15 0.121

Age Class 1 63 **1.27** 0.120 94 1.12 0.085

Class 2 237 **1.22** 0.066 37 0.78 0.151

Combined 300 **1.23** 0.058 131 1.02 0.075

___________________________________________________________________

The higher mean species richness at each level is highlighted in bold.

**Table S3.** NSpir helminth species richness (i.e., not Spirurida) in spiny mice

with or without *Protospirura muricola* by year, site, sex, and host age class.

_________________________________________________________________

*P. muricola* present *P. muricola* absent

_________________ _________________

Factor Level *n* Mean SEM *n* Mean SEM

__________________________________________________________________

Year 2000 32 1.09 0.158 21 **1.24** 0.194

2004 59 **1.66** 0.125 26 1.12 0.178

2008 58 0.97 0.139 38 **1.03** 0.144

2012 78 **1.21** 0.107 41 0.95 0.135

Site^1^ El Arbaein 57 **1.28** 0.143 55 0.85 0.111

Gharaba 89 0.99 0.090 23 **1.00** 0.189

Tlah 81 **1.51** 0.115 48 1.31 0.127

Sex Males 102 **1.13** 0.098 69 0.97 0.092

Females 125 **1.34** 0.090 57 1.16 0.132

Age Class 1 48 **1.29** 0.146 82 1.21 0.091

Class 2 179 **1.23** 0.075 44 0.77 0.137

Combined 227 **1.25** 0.066 126 1.06 0.078

___________________________________________________________________

The higher mean species richness at each level is highlighted in bold.

1. Data from Wadi Gebal have been excluded, because only one of 78 mice was infected with *P. muricola*, and only carried one worm.

**Table S4.** Quantitative covariance of species richness and worm burdens of non-spirurid (NSpir) helminth taxa with worm burdens of the Spirurida (SpirWB) or with those of *Protospirura muricola* (PmWB), as reflected in the linear regressions between the residuals of minimum sufficient models (MSMs).

_________________________________________________________________________________________________

Residuals of MSM

________________________________

Dependent variable Independent Pearson’s Adjusted slope (ß) ± SEM *t*^3^ DoF^3^ *P*^3^

Variable variable r^1^ r^2^

___________________________________________________________________________________________________

Species richness ^4^ SpirWB 0.160 0.0234 0.1585 0.04710 3.364 429 0.00084

Species richness PmWB 0.184 0.0310 0.1852 0.05292 3.500 351 0.00052

NSpir helminths SpirWB 0.136 0.0162 0.1326 0.04663 2.844 429 0.00467

NSpir helminths PmWB 0.143 0.0178 0.1439 0.05296 2.716 351 0.00693

NSpir nematodes SpirWB 0.113 0.0104 0.1035 0.04404 2.350 429 0.0192

NSpir nematodes PmWB 0.132 0.0146 0.1246 0.05000 2.491 351 0.0132

Oxyuroidea SpirWB 0.102 0.0081 0.0906 0.04274 2.119 429 0.0347

Oxyuroidea PmWB 0.115 0.0104 0.1052 0.04864 2.163 351 0.0312

Intestinal cestodes SpirWB 0.145 0.0187 0.0813 0.02683 3.030 429 0.0026

Intestinal cestodes PmWB 0.127 0.0133 0.0727 0.03034 2.398 351 0.0170

___________________________________________________________________________________________________

1. The sample size for all regressions that included SpirWB = 431, and those for PmWB = 353.

2. The r^2^ is for the regression, followed by the gradient of the slope and the SEM of this estimate.

3. Student’s *t,* degrees of freedom (DoF) and *P* values for test of whether the slopes differ from zero.

4. Species richness of non-spirurid helminths. For this dependent variable the MSM was based on Poisson errors. All the others were based on models with negative binomial error structures.

**Fig. S1.** Covariance of residuals from MSM for combined spirurid worm burdens and those of MSM for specified taxa among mice that were infected with at least one worm of each taxon

We first fitted MSM for each of the specified taxa and saved the residuals of these models. Then we selected records of mice that had at least one spirurid worm and at least one worm of the specified taxon. The figs. show the covariance and the 95% confidence limits.

A. *N*=226, *ß* = 0.154 ± 0.0731, *t*=2.112, *P*=0.0358, Adjusted *R*^2^ = 0.0152

B. *N*=191, *ß* = 0.146 ± 0.0750, *t*=1.944, *P*=0.0534, Adjusted *R*^2^ = 0.0144

C. *N*=179, *ß* = 0.162 ± 0.0760, *t*=2.134, *P*=0.0342, Adjusted *R*^2^ = 0.0196

D. *N*=50, *ß* = 0.085 ± 0.1233, *t*=0.691, *P*=0.4930, Adjusted *R*^2^ = -0.0108

**Fig. S2.** Covariance of residuals from MSM for *Protospirura muricola* worm burdens and those of MSM for specified taxa, among mice that were infected with at least one worm of each taxon.

We first fitted MSM for each of the specified taxa and saved the residuals of these models. Then we selected records of mice that had at least one *P. muricola* worm and at least one worm of the specified taxon. The figs. show the covariance and the 95% confidence limits. All records from Wadi Gebal have been excluded because *P. muricola* was extremely rare in that wadi.

A. *N*=174, *ß* = 0.164 ± 0.0895, *t*=1.831, *P*=0.0688, Adjusted *R*^2^ = 0.0134

B. *N*=145, *ß* = 0.212 ± 0.0902, *t*=2.350, *P*=0.0201, Adjusted *R*^2^ = 0.0305

C. *N*=134, *ß* = 0.195 ± 0.0928, *t*=2.103, *P*=0.0374, Adjusted *R*^2^ = 0.0251

D. *N*=38, *ß* = 0.012 ± 0.1370, *t*=0.088, *P*=0.9300, Adjusted *R*^2^ = 0.0275
